# Quantifying Biomolecular Binding Constants using Video Paper Analytical Devices

**DOI:** 10.1002/chem.201802394

**Published:** 2018-06-08

**Authors:** Benjamin S. Miller, Claudio Parolo, Valérian Turbé, Candice E. Keane, Eleanor R. Gray, Rachel A. McKendry

**Affiliations:** ^1^ London Centre for Nanotechnology University College London 17–19 Gordon Street London WC1H 0AH UK; ^2^ Division of Medicine University College London, Cruciform Building Gower Street London WC1E 6BT UK

**Keywords:** adsorption, nanoparticles, paper-based, protein–protein interactions, smartphones

## Abstract

A novel ultra‐low‐cost biochemical analysis platform to quantify protein dissociation binding constants and kinetics using paper microfluidics is reported. This approach marries video imaging with one of humankind's oldest materials: paper, requiring no large, expensive laboratory equipment, complex microfluidics or external power. Temporal measurements of nanoparticle–antibody conjugates binding on paper is found to follow the Langmuir Adsorption Model. This is exploited to measure a series of antibody–antigen dissociation constants on paper, showing excellent agreement with a gold‐standard benchtop interferometer. The concept is demonstrated with a camera and low‐end smartphone, 500‐fold cheaper than the reference method, and can be multiplexed to measure ten reactions in parallel. These findings will help to widen access to quantitative analytical biochemistry, for diverse applications spanning disease diagnostics, drug discovery, and environmental analysis in resource‐limited settings.

Today, a new generation of low cost consumer electronic‐based biosensors is emerging^1^, ^2^ with the potential to dramatically widen access to analytical chemistry capabilities in resource‐limited settings.^3^, ^4^, ^5^ This emerging field seeks to harness: mass manufactured sensors found within smartphones, such as cameras, to electronically capture test results; phone battery to power external devices; processing power to analyze results; screens to display results; and connectivity to transmit geo‐located results to central databases. There is increasing interest in the use of smartphones to detect results from lateral flow tests. Lateral flow tests, also known as microfluidic paper‐based analytical devices (μPADs), including 2D^6^ and 3D structures,^7^, ^8^, ^9^ and paper origami,^10^, ^11^, ^12^, ^13^ are opening up new capabilities for multiplexed analysis with small sample volumes and on‐test sample handling. The merits of μPADs are their compatibility with a broad range of chemical and biological molecules, low non‐specific interactions, low manufacturing cost (as little as $0.001^7^), portability, low sample volumes, safe disposal and power‐free fluid pumping, exploiting the natural capillarity of paper.^14^

To date, the use of smartphone cameras to interpret lateral flow tests has focused on individual still image end‐point readings to interpret paper‐based tests for diagnostics,^15^, ^16^, ^17^, ^18^, ^19^, ^20^, ^21^, ^22^, ^23^ chemical sensing,^24^, ^25^ and drug monitoring.^26^, ^27^ Cameras and smartphones have also been used with other microfluidic techniques to quantify biological reactions, such as the detection of nucleic acid sequences^28^ and *E. coli* detection using quantum dots.^29^ Video tracking of biological interactions has also been used for real‐time recording polymerase chain reaction amplification using a digital single lens reflex camera,^30^ glucose sensing with a mobile phone,^25^ and the use of a complementary metal‐oxide semiconductor image sensor to track the motion of sperm cells.^31^ Temporal surface plasmon resonance protein detection has also been demonstrated with a smartphone^32^ using a polydimethylsiloxane microfluidic device.

Here, for the first time in the literature, we progress beyond still images of lateral flow tests to video analysis in order to investigate whether dynamic ligand–receptor binding on μPADS follows the Langmuir Adsorption Isotherm Model,^33^ and whether μPADs could quantify fundamental biomolecular parameters, namely, the thermodynamic equilibrium dissociation constant, *K*_D_ and kinetic *k*_on_ and *k*_off_ rates. We overcome potential barriers associated with quantitative analysis on lateral flow tests cited in previous work, including sample volume limitations,^34^ color inhomogeneity,^2^ reproducibility issues,^35^ such as surface flow and inconsistent membranes,^2^ porous 3D surface, protein dissociation over long periods, and possible reaction‐limiting local sample depletion due to flow rate.

The ability to measure such fundamental chemical binding constants and kinetic reaction rates lies at the heart of chemistry and traditionally relies on access to sophisticated laboratory instrumentation, such as surface plasmon resonance^36^ and interferometry, used here as a gold‐standard reference method, typically using instruments costing in excess of £100 000. Other label‐free methods that also require specific instrumentation are dynamic light scattering^37^ and isothermal titration calorimetry.^38^ There are a variety of fluorescence‐labeling techniques such as fluorescence polarization,^39^ fluorescence correlation spectroscopy,^40^ total internal reflection fluorescence microscopy,^41^ and Förster resonance energy transfer.^42^ These methods all require fluorescence readout, such as a fluorescence microscope or spectrometer. In contrast, the method presented here is ultra‐low cost, simply requiring a digital camera/smartphone, giving equipment costs of just £500/£214 respectively, and per assay paper microfluidic strip and consumables costs of approximately £1.20 (Supporting Information (SI), Table S1).

Our low‐cost technique uses a simple set‐up consisting of a consumer camera or smartphone, a series of direct‐detection μPADs, and a 96‐well plate. Lines of antigen are immobilized on nitrocellulose paper strips. When the antibody‐functionalized gold nanoparticles (Ab‐AuNP) flow along the membrane, they bind to the test line (Scheme 1 a), generating a red‐color, the intensity of which is proportional to the number of gold nanoparticles (SI, Figure S1), and therefore the number of bound antibody‐antigens. The nitrocellulose strips are mounted together, with a large absorbent pad to prevent saturation, and dipped into a 96‐well plate, where each well contains a different concentration of Ab‐AuNP solution (Scheme 1 b). The camera videos the μPAD experiment running, as shown in Scheme 1 c. An excess solution volume is used in order to mimic an infinite solution (see SI, Figures S2–S5 for flow rate analysis). Scheme 1 d shows a series of video frames to show the temporal development of a μPAD test line. Video analysis (Wolfram Mathematica) is used to extract changes in colorimetric intensity, *I*. The pixel values are extracted and averaged across the width of the μPAD to reduce noise, creating a line profile along the strip. The peak height is then outputted as a function of time for each μPAD. Here, the green channel of the RGB color‐space is used to match the absorption peak of 20 nm AuNPs, but this can be tailored to the type of nanoparticles employed. Due to the timescales considered here, a sampling rate of 1 Hz is used; however, this could be increased up to 30 Hz to measure faster biochemical reactions.

**Scheme 1:**
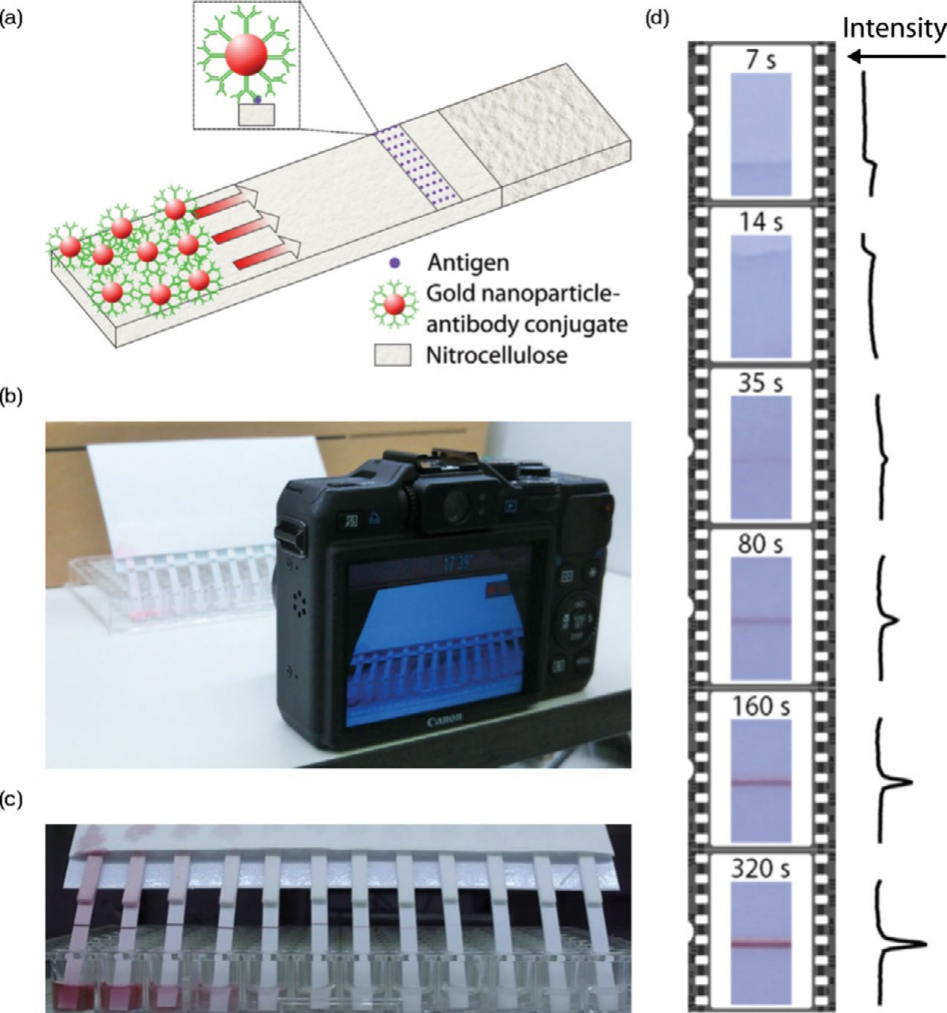
Temporal consumer‐electronic camera video analysis of μPADs. (a) A schematic of a direct‐detection μPAD. 20 nm gold nanoparticles (AuNPs), conjugated with an antibody of interest, flow up nitrocellulose strips, binding to a test line printed with antigen. This generates a red color (peak absorption 525 nm). (b) A camera captures the running of twelve video‐μPAD strips, allowing for parallel video processing. (c) A video frame of a set of video‐μPADs. A 96‐well plate is used for sample reservoirs, and an extended absorbent pad is used at the top to prevent saturation. Video‐μPADs are mounted together, allowing multiple strips to be started simultaneously, and immersed vertically in the solutions, ensuring the liquid moves along the strips only by capillary action. (d) An example series of video frames of a μPAD test line developing. Line profiles (right) show width‐averaged intensity along the strip.

Figure 1 a shows an example set of video‐μPAD time‐intensity plots to track the binding of a monoclonal antibody to the influenza hemagglutinin H5 antigen test line. A series of eight different Ab‐AuNP concentrations are measured (0.9 pM to 1.9 nm). The number of antibodies per AuNP, and antigen‐on‐strip concentration, are held constant. Time‐intensity plots are fitted to the exponential equation, shown below as Equation (1):

(1) $$I=I^{\infty }\left(1-e^{-k_{\mathrm{obs}}t}\right)$$

**Figure 1:**
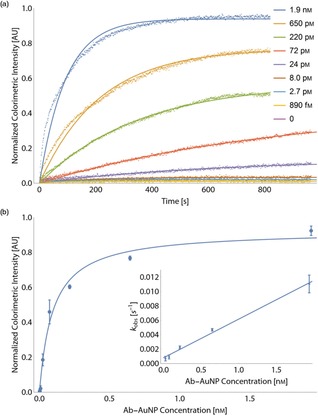
Applying Langmuir model to μPADs: (a) A typical series of intensity‐time binding plots for different concentrations of anti‐H5 Ab‐AuNPs binding to μPAD test lines functionalized with H5N1 hemagglutinin antigen. Raw data are shown as dots and exponential line fits (Equation (1)) are shown as solid lines. (b) Langmuir plot of extracted infinite test line intensity values, *I* ^*∞*^, from (a). Experiment performed in triplicate and results shown as the mean with error‐bars representing the standard deviation. The data are fitted to the Langmuir model (Equation (2)) (solid line). Inset is a graph of the observed reaction rate, *k*_obs_, plotted against the concentration of Ab‐AuNP solution. The gradient of the linear fit (solid line) corresponds to the reaction on‐rate, *k*_on_, and the *y*‐intercept, the reaction off‐rate, *k*_off_ (Equation (3)).

where *I* is intensity (test line peak height), $I^{\infty }$ is the equilibrium intensity value, *k*_obs_ is the fitted observed binding rate, and *t* is time. As t→∞ , $I\to I^{\infty }$ . The $I^{\infty }$ values are determined for each concentration of analyte, and fitted to a Langmuir model shown below as Equation (2), 33 (Figure 1 b):

(2) $$I^{\infty }=a\cdot C/\left(K_{D}+C\right)$$

where *a* is the saturation intensity when all available binding sites are occupied, and *C* is Ab‐AuNP concentration.

To determine the *k*_on_ and *k*_off_ rates, the values of *k*_obs_ are extracted from the fits of Equation (1) and plotted against *C*. They are then fitted to following the relationship in order to extract *k*_on_ and *k*_off_, shown below as Equation (3):

(3) $$k_{\mathrm{obs}}=k_{\mathrm{off}}+k_{\mathrm{on}}\cdot C$$

An example of this is shown in Figure 1 b (inset).

Our results show strong agreement between the binding kinetics measured by video analysis and the Langmuir model.

We then apply video analysis to five different antibody‐antigen pairs (SI, Table S2). In parallel, benchmarking studies are performed with the same proteins using a FortéBio Octet RED96 benchtop interferometer. The *K*_D_ fits shown in Figure 2 a demonstrate strong agreement between video‐μPADs (solid lines) and interferometry (dotted lines). This is further demonstrated in Figure 2 b, where the *K*_D_ values measured by video‐μPADs and interferometry show a linear relationship with a gradient of 1.1 (standard error 0.032), and an adjusted R^2^ value of 0.996. For all raw data and fits, see SI, Figures S6, S7, and Table S3. The estimated concentration of antibodies in video‐μPAD assays assumes that all antibodies bind to AuNPs in an active, available conformation, equating to ≈15 antibodies per AuNP. We note that the measured *k*_on_ and *k*_off_ values differ from those measured by interferometry, although the relationship *K*_D_=*k*_off_/*k*_on_ still holds (SI, Figures S8 and S9). This interesting result highlights the value of quantifying antibody‐antigen reaction kinetics on μPADs in order to optimize diagnostic test performance to achieve fast kinetics and a strong *K*_D_, giving a sensitive, rapid test with low‐sample volume.

**Figure 2:**
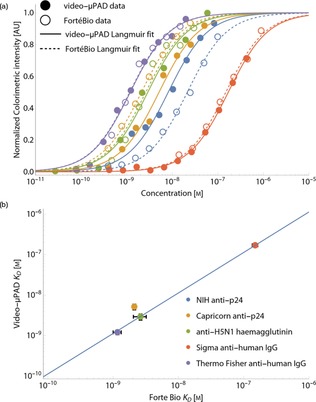
Quantifying *K*_D_ with video‐μPADs. (a) A comparison of measured *K*_D_ values on video‐μPADs versus the reference FortéBio Octet interferometer benchtop device for five different antibody‐antigen pairs. The filled circles and solid lines correspond to the experimental values and fits on video‐μPADs. The concentration is corrected by a factor of 30.6 for the number of binding sites on each AuNP (two paratopes per antibody). The hollow circle and dotted lines show the experimental values and fits of the same antibody–antigen pairs measured by interferometry. (d) Direct comparison of the *K*_D_ values measured on video‐μPADs (corrected for the number of antibodies on each AuNP) and by interferometry. The solid line is a linear fit with a *y* intercept of (0, 0), giving a gradient of 1.1 with adjusted *R* ^2=^0.996.

A proof‐of‐principle of video‐μPADs using a smartphone is shown with an LG Nexus 5 (Figure 3 a). The performance is compared to the Canon Powershot G15 camera for a model anti‐human IgG Fc‐human IgG interaction (Figure 3 b and SI, Figure S10). No significant difference is found between the resulting *K*_D_ values (two‐tailed *t*‐test gives *p*‐value of 0.22, *t*‐value of 1.2, degrees of freedom=52), confirming the LG Nexus 5 smartphone can be used for *K*_D_ measurements.

**Figure 3:**
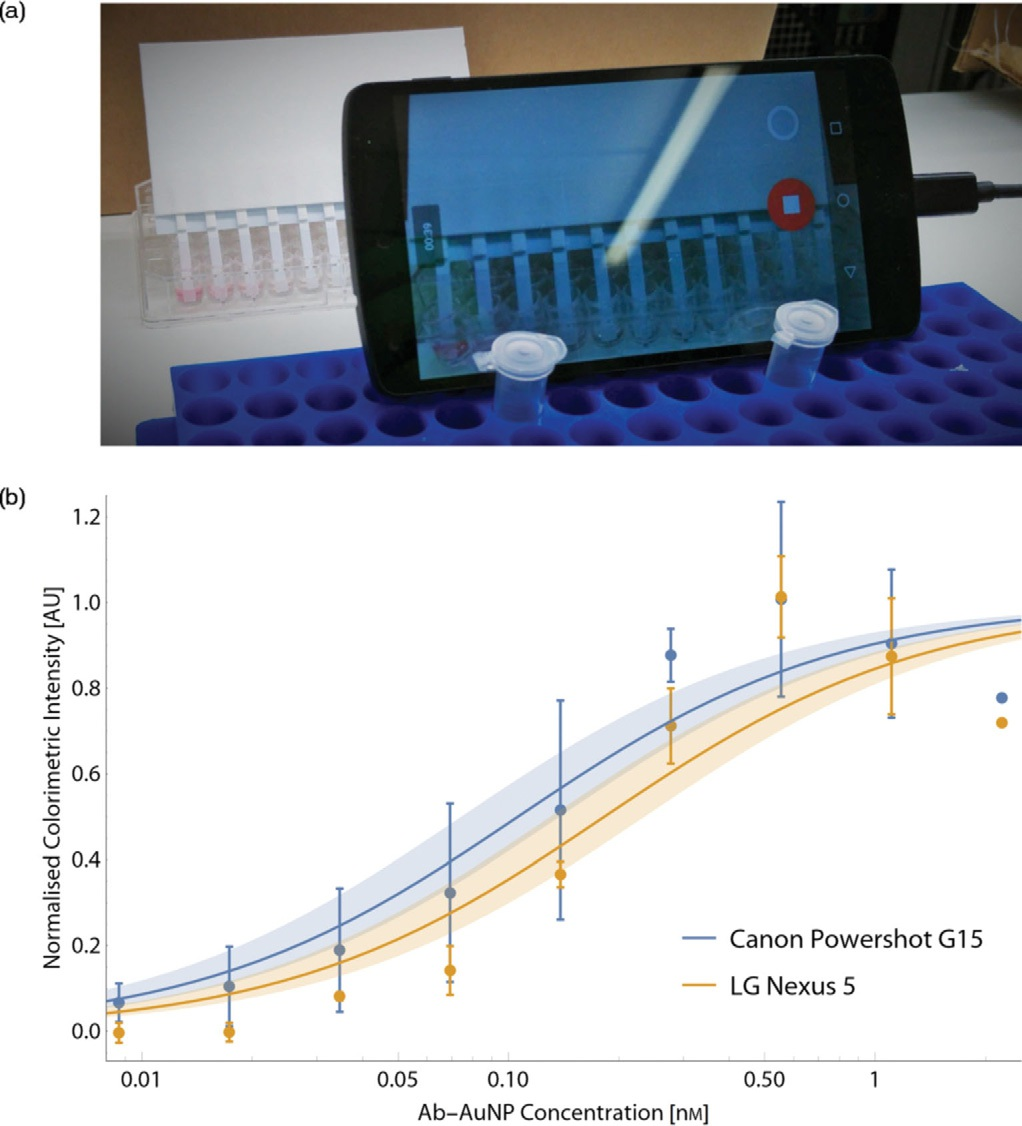
Measuring *K*_D_ using a smartphone. (a) A photograph of a smartphone video recording the running of direct‐detection μPADs. (b) A comparison of two Langmuir curves calculated from the same samples recorded by the Canon Powershot G15 camera, and LG Nexus 5 smartphone. The data points are the fitted infinite‐time intensity values, with error bars the standard deviation from triplicate measurements. The shaded regions show the standard errors of the *K*_D_ values.

Building on our work with single antibody–antigen pairs, we then sought to investigate whether video‐μPADs could measure multiple antibody–antigen interactions simultaneously. This could be useful for antibody and drug screening to quantify multiple antibodies’ binding affinities to a single target. Therefore, in contrast to the above, the AuNPs are functionalized with the antigen, and the antibody is spotted on the μPADs. The proof‐of‐concept is shown in Figure 4. An array of ten antibody spots is deposited on each μPAD. Figure 4 a shows a filmstrip of a multiplex video‐μPAD developing over time, with a heat map of pixel values shown below. This is translated into the time‐intensity graph in Figure 4 b showing nine multiplex video‐μPADs—eight different antigen‐AuNP concentrations and a buffer control. Each μPAD′s ten spots are plotted overlaid for each concentration. The low variances between spots illustrates that the kinetics are independent of spot position. Here, identical antibody–antigen combinations are used as a proof‐of‐concept. In future, each spot could be a different capture ligand. We show that the *K*_D_ measured from singleplex and multiplex video‐μPADs are not significantly different (two‐tailed *t*‐test gives *p*‐value=0.25, *t*‐value=1.1, degrees of freedom=96), validating this reversed orientation (see SI, Figure S11).

**Figure 4:**
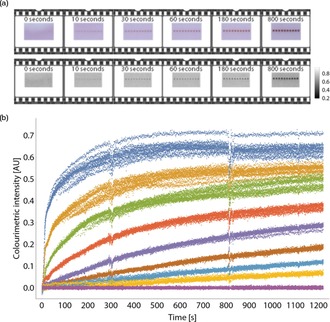
Towards highly multiplexed video‐μPAD kinetic binding assays. (a) The development of an example region of interest from a multiplexed μPAD as a function of time over a period of 800 seconds. The filmstrip below is a “heat map” of the pixel values from the corresponding raw images. (b) A plot to show the intensity–time response to eight different concentrations of antigen‐AuNPs (0.027–3.5 nm) and a buffer control is shown. Each strip has ten individual spots, measured independently, and the data overlaid. Here, as proof‐of‐principle, all ten spots on a single strip are functionalized with the same antibody–antigen interaction, the high reproducibility demonstrates the feasibility of multiplexed analysis.

Herein we harness consumer electronic video imaging for low‐cost μPADs, creating an accurate platform for measurement of antibody–antigen dissociation constants. Our approach does not require expensive, complex laboratory‐based equipment, simply using a camera or smartphone. The measured video‐μPAD *K*_D_ values for five antibody–antigen pairs show excellent agreement with a reference benchtop interferometer (Figure 2 b, adjusted *R* ^2^=0.996). The assumptions and justifications for using the Langmuir model are discussed in SI Discussion and Table S4. We demonstrate that a smartphone can measure *K*_D_ values, and create a multiplex platform to detect multiple ligand–receptor interactions in parallel. Although AuNP labeling is used here, future assays could employ a label‐free competitive inhibition format.

The low‐cost of video‐μPADs is a major advantage over expensive benchtop instrumentation. SI Table S1 lists the cost, size, and weight of video‐μPAD instrumentation compared to the FortéBio Octet RED96, showing that video‐μPAD equipment costs are around 250–580 times cheaper, making it much more accessible to academic laboratories and resource‐limited settings. Low‐end smartphones are amenable to this application, since neither high camera resolutions nor large processing power are needed. Moreover, video‐μPADs require 64‐fold lower amounts of capture reagents (SI Table S1), advantageous for early stage discovery projects requiring biophysical characterization of reagents, where the quantities of material may be limited.

In our study, video analysis is performed off‐device; however, this could be performed in real‐time, even on a low‐cost smartphone, with a capture rate of 1 Hz. In future, automatic strip detection by traditional image processing or machine learning would make the method more user‐friendly.

To close, this technique allows low‐cost, quantitative, multiplexed analysis and is generalizable to a wide range of biological and chemical ligand–receptor interactions, with many potential applications in analytical chemistry, biomedicine, forensics, and environmental analysis.

## Conflict of interest

The authors declare no conflict of interest.

## Supporting information

As a service to our authors and readers, this journal provides supporting information supplied by the authors. Such materials are peer reviewed and may be re‐organized for online delivery, but are not copy‐edited or typeset. Technical support issues arising from supporting information (other than missing files) should be addressed to the authors.

Supplementary

Supplementary
