# Supplementary material for: Quantifying Biomolecular Binding Constants using Video Paper Analytical Devices
Source: Chemistry. 2018 Jun 8;24(39):9783–7. doi: 10.1002/chem.201802394 (PMC6055620; doi:10.1002/chem.201802394)
Supplement: Supplementary file 1 — Supplementary [file CHEM-24-9783-s001.pdf]

CHEMISTRY
A European Journal

Supporting Information

Quantifying Biomolecular Binding Constants using Video Paper Analytical Devices

Benjamin S. Miller,[a, b] Claudio Parolo,[a] Valérian Turbé,[a, b] Candice E. Keane,[a, b] Eleanor R. Gray,[a] and Rachel A. McKendry*[a, b]

chem_201802394_sm_miscellaneous_information.pdf
chem_201802394_sm_movie.gif

Author Contributions

B.M. Conceptualization: Equal; Data curation: Lead; Formal analysis: Lead; Investigation: Lead; Methodology: Lead; Writing – original draft: Lead; Writing – review & editing: Lead
C.P. Investigation: Supporting; Methodology: Supporting; Supervision: Supporting; Writing – review & editing: Supporting
V.T. Formal analysis: Supporting; Investigation: Supporting; Methodology: Supporting; Writing – review & editing: Supporting
C.K. Resources: Supporting; Writing – review & editing: Supporting
E.G. Resources: Supporting; Writing – review & editing: Supporting
R.M. Conceptualization: Lead; Funding acquisition: Lead; Project administration: Lead; Supervision: Lead; Writing – original draft: Supporting; Writing – review & editing: Equal.

Supporting Information

Costings 2
Experimental Methods 2
Video-μPADs 2
FortéBio Interferometry 3
Colorimetric measurement of AuNPs 3
Characterisation of capillary flow 3
Flow Rate and Evaporation 4
Wetting phase: fluid front propagation 5
Langmuir adsorption model fitting 6
Association (k_{on}) and dissociation (k_{off}) rates 11
Comparison of k_{off}/k_{on} with K_D from Langmuir fit 11
k_{on} and k_{off} measured on video-μPADs and FortéBio Octet 12
Comparison of video-μPAD measurements with camera and smartphone 13
Multiplexing video-μPADs 14
Supporting Discussion: Assumptions K_D and Rate Limiting Factors 14

Costings

	Benchtop Biolayer Interferometer	Digital Camera Video-µPADs	Smartphone Video-µPADs
Instrument Model	FortéBio Octet Red96	Canon Powershot G15 (12MP, F1.8-2.8, 28-140mm)	LG Nexus 5 (8MP, F2.4, 30mm)
Dimensions (cm)	47 x 43 x 53	10.7 x 7.6 x 4.0	14 x 7 x 1
Weight	24.5kg	0.35kg	0.13kg
Approximate cost of reagents per assay *	96-well plate: £1.2<br>Sensors: £36.67<br><br>Capture ligand: £0.15 (32 µg)<br><br>Total: £38.02	96-well plate: £0.33<br>Lateral flow: £0.47<br>AuNP: ~£0.40<br><br>Capture ligand: £0.0024 (0.5 µg)<br><br>Total: £1.20	96-well plate: £0.33<br>Lateral flow: £0.47<br>AuNP: ~£0.40<br><br>Capture ligand: £0.0024 (0.5 µg)<br><br>Total: £1.20
Approximate Cost	£125,000**	£500	£214

Table S1. Comparison of device and reagents costs for video-µPADs and FortéBio Octet Interferometer. * Costs based on FortéBio quote, Millipore quote and Sigma prices; **cost based on price enquiry. All costs based on low volume lab purchases.

Experimental Methods

Video-µPADs

2D-µPAD strips used for antibody-antigen binding kinetics are Millipore Hi-Flow™ Plus Membranes. Nitrocellulose membranes (HF180 and HF240) are mounted on membrane backing card (Millipore). Cellulose absorbent pads are mounted at the top of the strips. The antigen of interest is printed in a line near the top of the nitrocellulose using a Micropdrop Autodrop Professional Positioning System AD-P-8000, or spotted with a pipette. Antigen is printed at 0.21 mg/mL in water with 0.79 mg/mL BSA. For lower affinity antibodies, antigen concentration is increased to 0.42 mg/mL to increase signal, with BSA concentration decreased to maintain 1 mg/mL overall protein concentration. Strips are cut to a width of 3 mm using a guillotine.

Antibodies of interest are conjugated to 20 nm AuNPs purchased from BBI (EM.GC20). 4 µg of antibody are added to 1.5 mL of AuNPs at 1.16 nM with shaking at 650 rpm for 25 minutes at 25 °C in a thermoshaker (Grant-bio). 100 µL BSA in deionized water (1 mg/mL) is mixed in for blocking, and left for a further 25 minutes shaking at 650 rpm at 25 °C. The AuNPs are then centrifuged at 14000 rpm (18407 × g), 4 °C for 20 minutes (Eppendorf Centrifuge 5424 R), the supernatant removed, and the conjugated AuNPs re-suspended in "running buffer" (1x PBS with 5% BSA and 0.05% Tween-20). Serial dilutions of Ab-AuNP are prepared in running buffer and pipetted into a 96-well ELISA plate along with a blank (running buffer).

Twelve strips are stuck to rigid backing card at the spacing of a 96-well ELISA plate, and a further large absorbent pad added at the top to absorb excess solution. The strips are placed in the 96-well ELISA plate containing the running solutions, which migrated up the strip and into the absorbent pad by capillary action. This process is filmed using a standard digital camera (Canon Powershot G15), and the resulting video files used to quantify antibody-antigen binding by temporal colorimetric analysis. For smartphone measurements, an LG Nexus 5 is used, running the Open Camera app.

Video analysis is performed in Wolfram Mathematica. Frames are imported at a rate of 1Hz, and the test line of the twelve strips is quantified by taking the intensity of the green channel in the RGB colour-space, averaging pixel values across the width of the strip to reduce noise. Strips are normalized to the intensity value when the fluid front has just crossed the test line, and the blank (running buffer) subtracted to account for ambient light variations between experiments. Uniform lighting between strips in the same experiment is achieved using lab lighting. The resulting time-intensity curve at each concentration is fitted as described.

All fitting is performed using the NonlinearModelFit function, using NMinimize to find a global minimum, by the differential evolution method. Outliers are identified by having simultaneously infinite-time intensity values that are

higher than other values and k_{obs} values lower. They are excluded from the k_{obs} fit. The outliers are refitted to exponentials, but with the value of k_{obs} constrained to be larger than or equal to the value taken from the k_{obs} fit.
The proteins used are:

Protein	Manufacturer	Catalogue Number
HIV-1-p24 antigen	Aalto Bio Reagents	AG6054
CP-HIV-1/2 monoclonal, purified	Capricorn	HIV-018-48303
NIH-3537 (National Institutes of Health)	NIH AIDS Reagent Program	3537
Mouse Monoclonal Antibody to Influenza A Virus H5N1 (Avian Flu) HA	Sino Biological	11048-MM01
Influenza A H5N1 (A/turkey/Turkey/1/2005) Hemagglutinin / HA Protein (His Tag)	Sino Biological	11061-V08H1
IgG from human serum	Sigma	I2511
Mouse anti-Human IgG Fc Secondary Antibody	Thermo Fisher Scientific	MA1-10378
Anti-Human IgG (Fc specific)–Biotin antibody, Mouse monoclonal	Sigma	B3773

Table S2. Summary of proteins used and manufacturer information.

FortéBio Interferometry

The benchtop FortéBio Octet RED96 “Bio-layer” Interferometer (Pall FortéBio LLC) is used as a gold standard for quantifying binding kinetics. Sensors used are anti-mouse (AMC) coated – the mouse antibody is immobilized to the sensor; and amine reactive (AR2G) – using a standard NHS-EDC protocol to covalently immobilize biomolecules to the sensor. 1x “kinetics buffer” (Pall FortéBio LLC) is used for association and dissociation.
Raw data files output from the FortéBio are imported into Wolfram Mathematica, and the method described above is used to quantify binding constants.

Colorimetric measurement of AuNPs

The principle of colorimetry on paper is verified by printing 25 wax “wells” onto Whatman Chromatography paper. They are then heated on a hot plate to melt the wax through the thickness of the paper, creating hydrophobic barriers. Different amounts of AuNPs (0.25 – 2 µL) are then pipetted into these “wells” and imaged with a Canon Powershot G15 camera. The colorimetric intensities are extracted and plotted against number of AuNPs per well. Figure S1 shows that there is a linear relationship between the number of AuNPs per unit area, and the colorimetric intensity. Therefore, the colorimetric intensity can be used to quantify the number of AuNPs bound on paper.

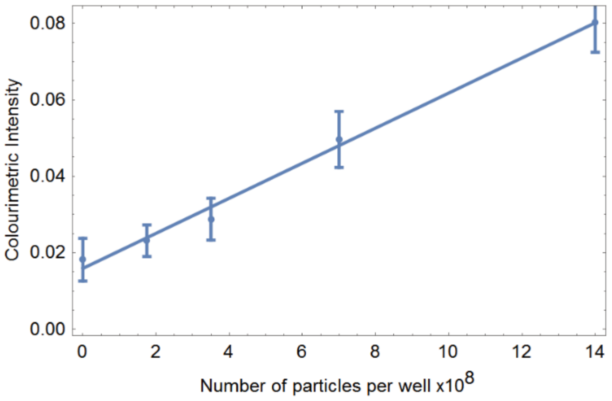

Figure S1. Colorimetry on paper. The colorimetric intensity (green channel of RGB colour-space) is plotted against number of particles per well (wells have equal areas). The solid line shows a linear fit, indicating that colorimetric intensity is linearly related to the number of particles per well (adjusted R^2 value = 0.990).

Characterisation of capillary flow

Flow Rate and Evaporation

Here, two hypotheses are tested. Firstly, that the flow rate is constant, following Darcy flow in the fully wetted phase,[1] (when the fluid has reached the top of the strip). For this purpose, a linear relationship is first confirmed between the volume of an Ab-AuNP solution and optical density (OD) measured with a plate reader. Different amounts of Ab-AuNP complexes in running buffer (PBS, 5% BSA, 0.05% Tween 20) are pipetted into wells of a 96-well plate. The absorption at 525nm is measured with a plate reader (Molecular Devices SpectraMax i3x). Each measurement is repeated five times. The results, shown in Figure S2a, show a linear relationship between Ab-AuNP solution volume and OD: $OD = 0.0035 * V - 0.0045$, where V is volume in µL (adjusted R^2 = 0.995).This relationship is subsequently used to convert absorption measurements into volumes.

To investigate flow rate, 96-well plate wells are filled with the same volume of identical Ab-AuNP solution in running buffer. Strips are then placed in the wells for different amounts of time. One well (flow time = 0) does not have a strip. After the strips are taken out, the remaining solution volume is measured using a spectrophotometer as described above. The experiment is performed in triplicate for each flow time, and measurements taken six times. The volume left is subtracted from initial volume to get solution volume flowed. This is plotted against flow time in Figure S2b. The remaining solution concentrations are measured using a NanoDrop One to check for any change (absorbance at 525nm), due to either the flow or evaporation. The constant gradient in Figure S2b shows a constant volumetric flow rate, as expected for Darcy flow, at 1.4 µL/min. The two points at flow time = 0 (strips not dipped into these wells) are one left at room temperature for 30 minutes, and one pipetted out after 30 minutes to compare evaporation. The measured volumes are not significantly different (p-value = 0.78, t-value = 0.30, degrees of freedom = 4) showing that, at these time scales, evaporation is negligible.

This is confirmed in Figure S2c, where the concentration in the wells is not significantly different (ANOVA p-value = 0.832, F-ratio = 0.538, degrees of freedom = 32), showing no change in reservoir concentration due to flow or evaporation.

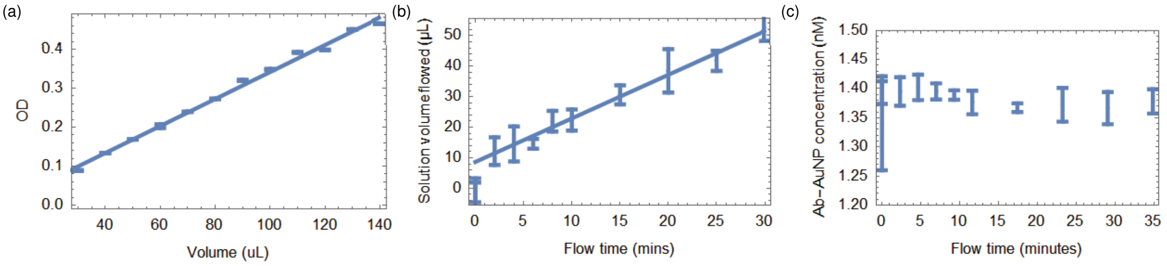

Figure S2. Flow rate analysis. (a)The relationship between the volume of Ab-AuNP solution and the absorption (OD) at 525nm. The error bars show the standard deviation of five measurements with a plate reader. The fitted linear regression has an adjusted R^2 value of 0.995. (b) The solution volume flowed is plotted against flow time. Each flow time is repeated in triplicate, and each well is measured six times. The mean of the six measurements is used. The error bars show the standard deviation between the triplicate experiments, giving a linear relationship with an adjusted R^2 value of 0.985, and a volumetric flow rate (gradient) of 1.4 µL/min. The well corresponding to the two points at flow time t = 0 do not have a strip in them. They represent: (1) left at room temperature for 30 minutes and (2) added at the end for comparison. They are both lower than expected because there is a small amount of fluid lost on the surface of the strip when they are removed, due to surface tension. Therefore, these two points are not included in the linear fit. There is no significant difference between the points (p value = 0.78), showing that over the time scales considered in this paper, evaporation is negligible. (c) The concentration of the solution in each well of the 96-well plate, showing no significant differences between wells (ANOVA p-value = 0.832, F-ratio = 0.538, degrees of freedom = 32). This indicates that there is no change in solution concentration over the measured period, due to either flow or evaporation.

Wetting phase: fluid front propagation

Video analysis is used to characterise the fluid propagation of the AuNP solution run up the strips during the wetting phase (when the strip is in transition from dry to wet). There is a nascent buffer front, closely followed by an AuNP front that initially travels more slowly. Figure S3 shows the AuNP front behind the buffer front. See also Supporting Video 1.

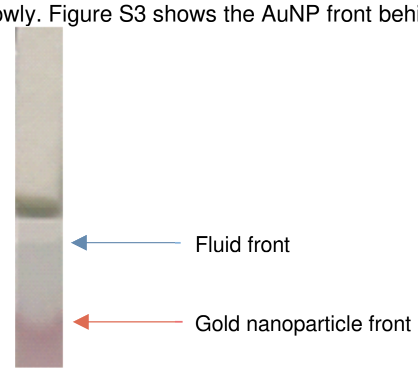

Figure S3. Fluid propagation of AuNP solutions on 2D-μPADs. A clear fluid front travels initially, then a red AuNP front. At the top of the strip is an absorbent pad.

Video analysis is use to characterize the flow of the two fronts. The clear fluid front appears as a step function in the intensity profile, so interpolation followed by numerical differentiation gives a moving delta function, which is tracked along the strip. The distance travelled by the fluid front squared, L^2, is plotted against time in Figure S4. Flow during the wetting phase follows the Washburn equation:[1,2]

$$L^2 = \frac{\gamma D t}{4\mu}$$

where L is the fluid front distance travelled, t is time, D is average pore diameter, γ is the effective surface tension, and μ is the viscosity.

The experimental results follow the Washburn equation, suggesting that the paper has consistent pore sizes, and gravitational forces are negligible.

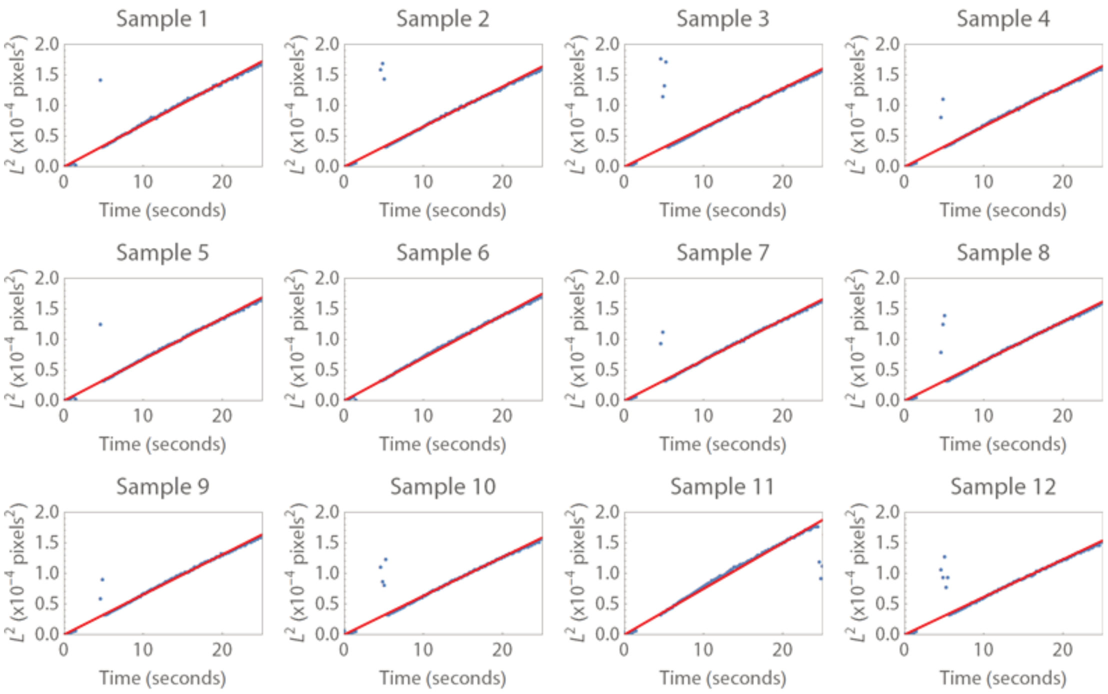

Figure S4. Buffer fluid front propagation. The twelve samples are run at different concentrations of AuNPs, including a blank (Sample 12) which is buffer alone. Plots of L^2 (distance travelled along the strip squared) against time show a linear relationship, following the Washburn equation. Adjusted R^2 values are between 0.95 and 1.0. The average gradient is 665 pixels^{2}s^{-1} with a standard deviation of 35.1 pixels^{2}s^{-1}.

The AuNP front travels more slowly, but still follows the Washburn equation, as shown in Figure S5. The smaller gradient reflects the slower flow. We show above (Figure S2) that the concentration of the reservoir does not change, so during fully wetted flow the flow rate of the Ab-AuNP is the same as the buffer. This is governed by the equation for Darcy flow: $Q = \frac{\kappa A \Delta p}{\mu L}$,[3] where κ is the permeability of the paper, A is the cross sectional area, Δp is the pressure difference along the flow direction, μ is the viscosity and L is the length of the channel. This shows that the viscosity is not different between the Ab-AuNP flow and buffer, and as the pore size, D, is constant, the surface tension, γ must vary for the AuNPs, slowing the initial flow, but not affecting fully wetted flow (Figure S2). In this range, the changing AuNP concentration does not affect flow rate.

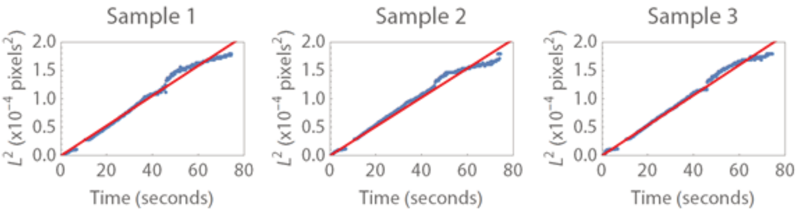

Figure S5. AuNP fluid front propagation. The AuNP front is tracked in three strips at three different Ab-AuNP concentrations (4.0, 2.0, 1.0 nM). L^2 is plotted against time, following the Washburn equation. Adjusted R^2 values are between 0.995-0.997. The average gradient is 241 pixels^{2}s^{-1} with a standard deviation of 4.31 pixels^{2}s^{-1}.

Langmuir adsorption model fitting

This section shows the raw data, and model fitting using video-µPADs. The following Supplementary Figures 6 and 7 show how the raw data is fitted to extract K_D, k_{on} and k_{off} values for video-µPADs and FortéBio Octet respectively. The raw data (normalized colorimetric intensity for video-µPADs and fractional binding for interferometry) is extracted by video analysis and plotted against time. Concentrations are chosen to be ~0.1-10x the K_D. These data are fitted to exponential equations to extract the equilibrium intensity values (I^{∞}) for each concentration. Equilibrium intensity values are plotted against concentration and fitted to a Langmuir plot. The exponential rate constants are also plotted against

concentration, and the resulting linear fit is used to extract k_{on} and k_{off}. The concentrations shown in Supplementary Figure 6 are Ab-AuNP concentration, rather than antibody concentration.

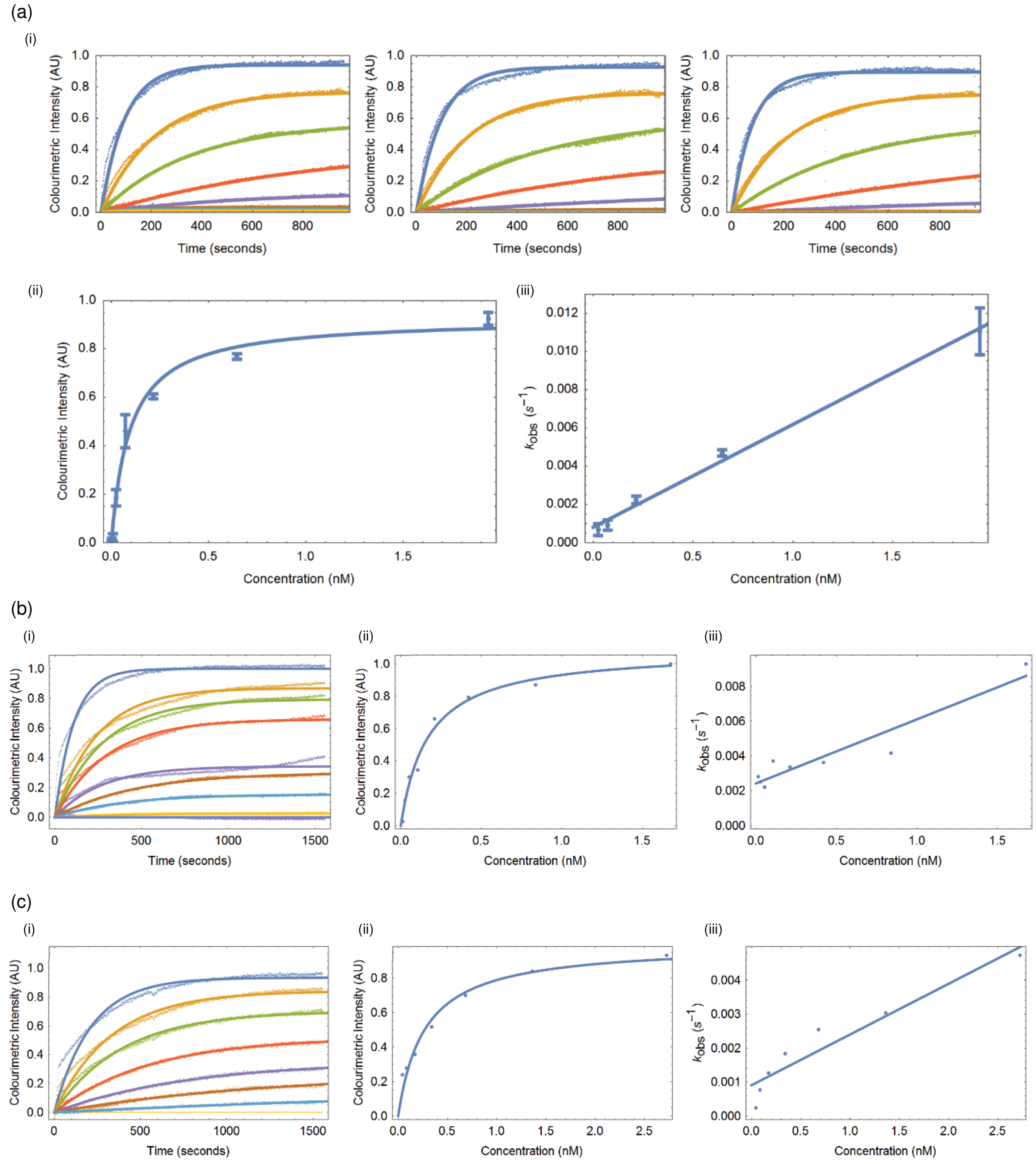

(d)

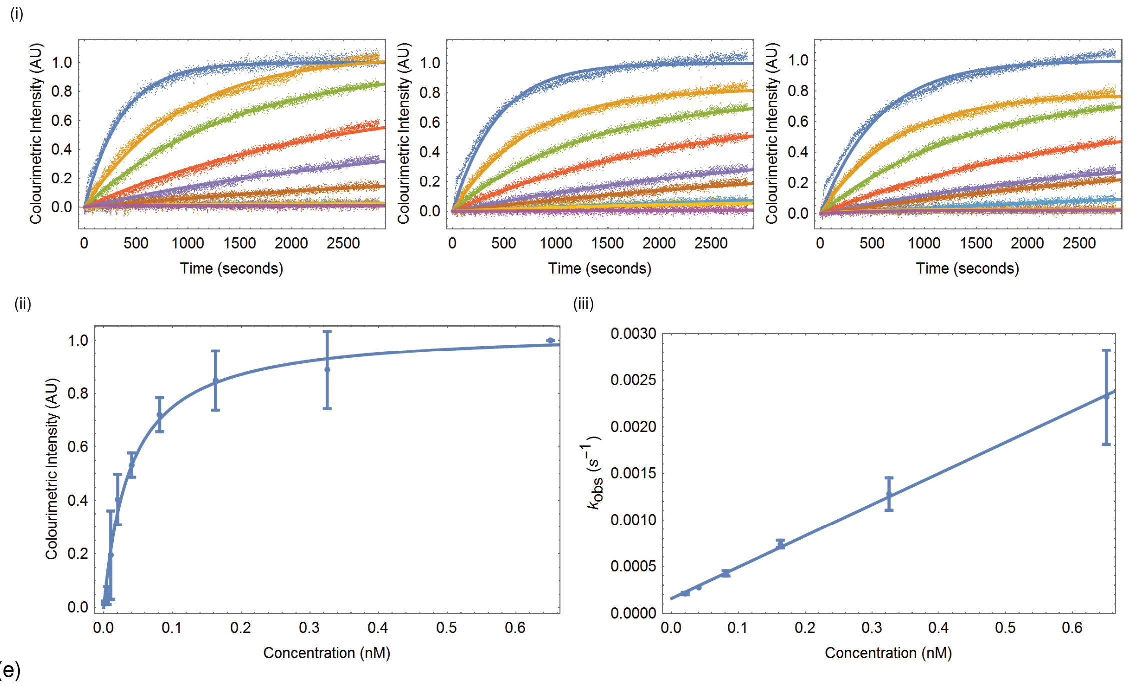

(e)

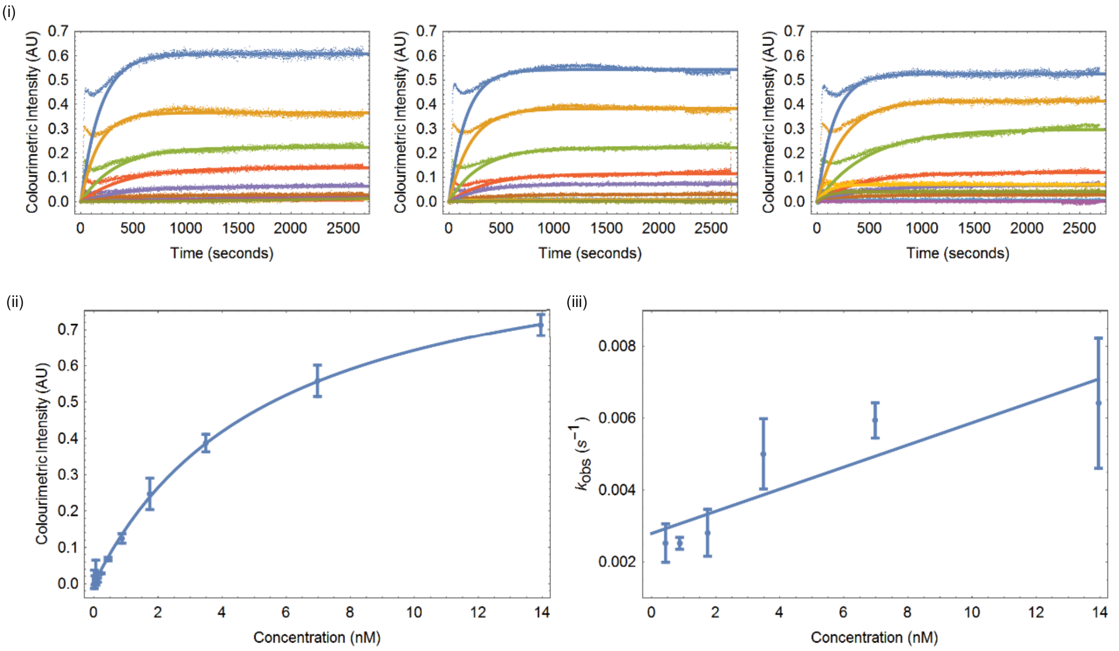

Figure S6. Video-μPAD raw data and model fitting. In all cases, a negative control (running buffer) is subtracted from each set of time-intensity curves. (a) Anti-H5 (anti-hemagglutinin) binding to H5 hemagglutinin. Ab-AuNP concentrations range from 0.0027 – 1.9 nM (n=3). (b) CP-HIV-1/2 binding to HIV-1 p24. Ab-AuNP concentrations range from 0.0016 – 1.7 nM (n=1). (c) NIH-3537 binding to HIV-1 p24. Ab-AuNP concentrations range from 0.043 – 2.7 nM (n=1). (d) Thermo Fisher Scientific Mouse anti-Human IgG Fc binding to human IgG. Ab-AuNP concentrations range from 0.0025 – 0.65 nM (n=3). (e) Sigma Monoclonal Anti-Human IgG (Fc specific)-Biotin binding to human IgG. Ab-AuNP concentrations range from 0.014 – 14 nM (n=3). In each part, (i) shows the raw data as dots, with exponential fits shown as solid lines; (ii) shows the Langmuir plot of fitted infinite-time intensity values (equilibrium intensities) against concentration. The fitted Langmuir curves are shown as solid lines; (iii) shows the observed rates, k_{obs} plotted against concentration. The solid lines show linear fits, where the gradients give k_{on} and the y-intercepts, k_{off}.

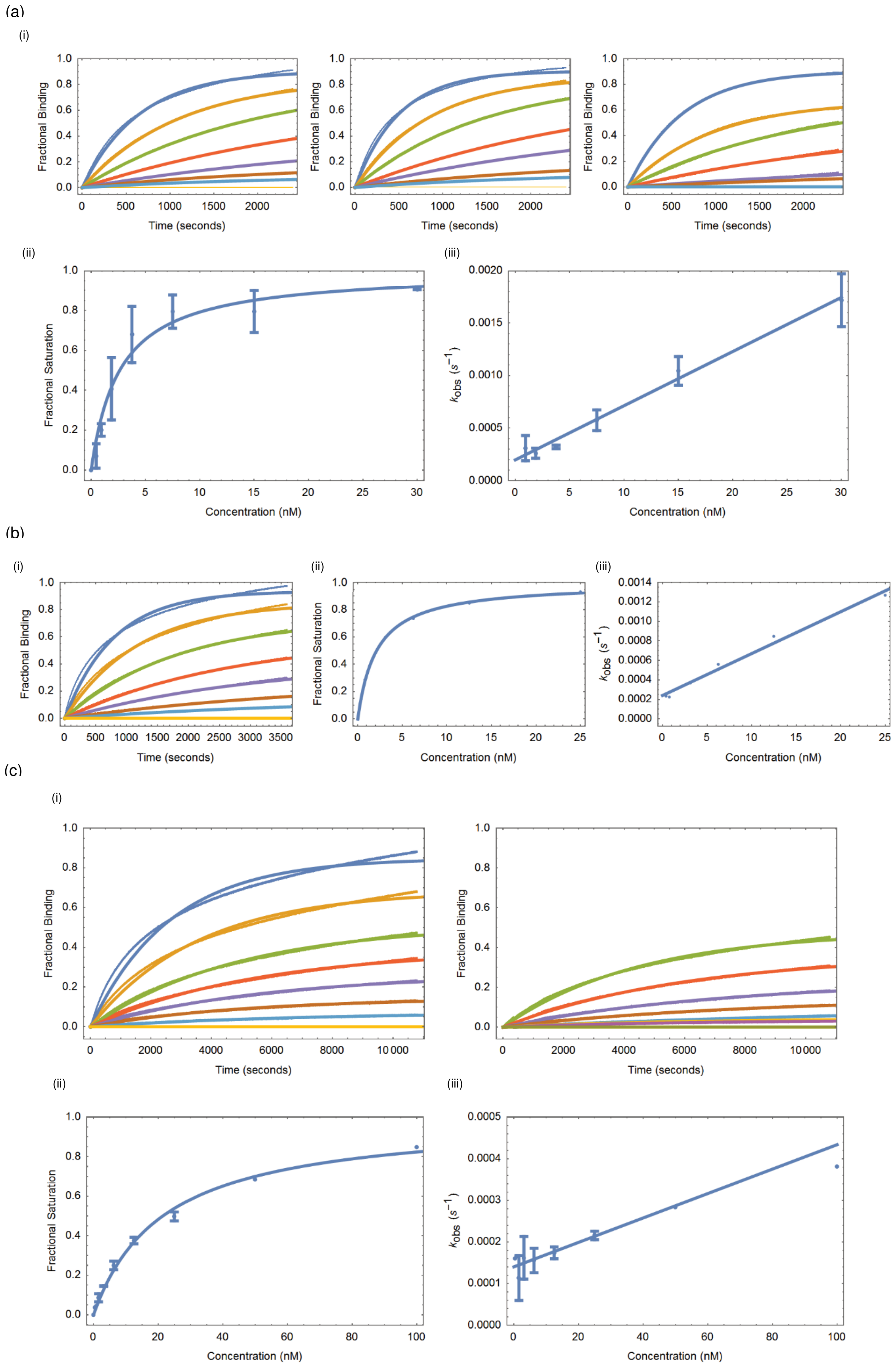
(a)
(i)
Fractional Binding
Time (seconds)
(ii)
Fractional Saturation
Concentration (nM)
(iii)
k_{obs} (s^{-1})
Concentration (nM)
(b)
(i)
Fractional Binding
Time (seconds)
(ii)
Fractional Saturation
Concentration (nM)
(iii)
k_{obs} (s^{-1})
Concentration (nM)
(c)
(i)
Fractional Binding
Time (seconds)
(ii)
Fractional Saturation
Concentration (nM)
(iii)
k_{obs} (s^{-1})
Concentration (nM)

(d)

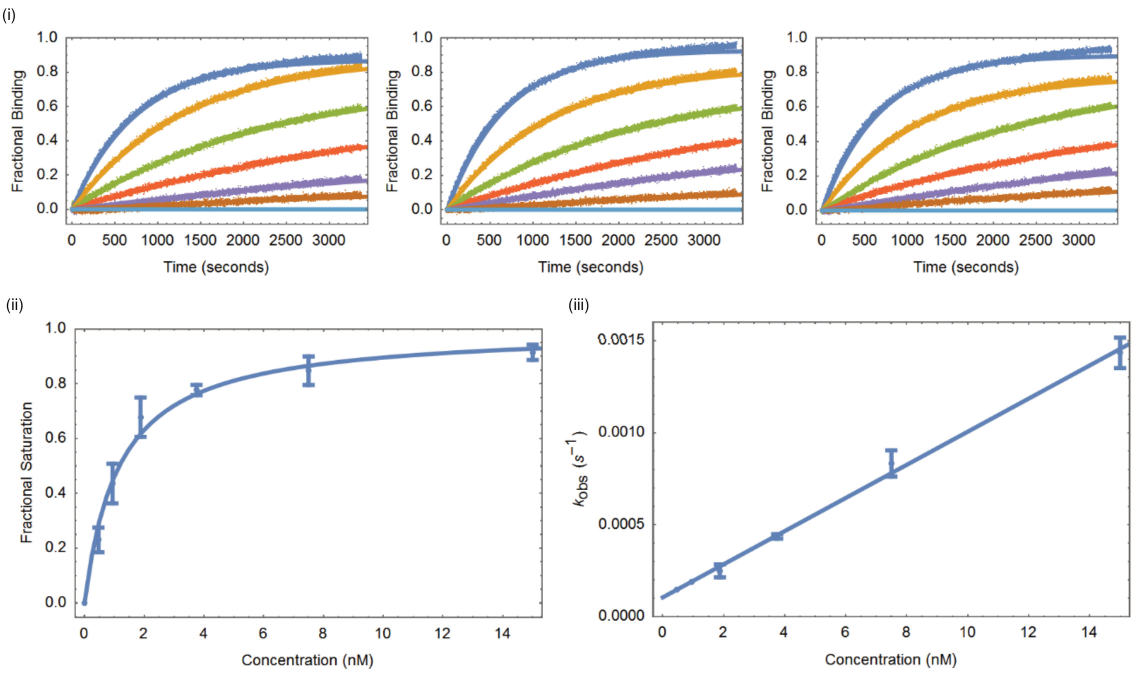

(e)

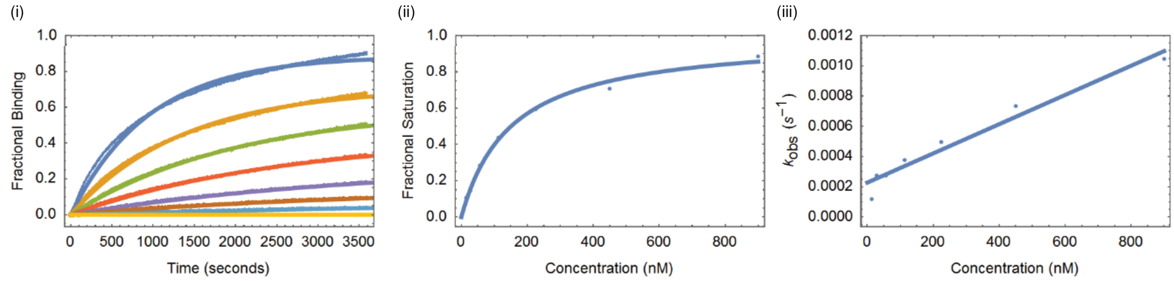

Figure S7. FortéBio Octet raw data. In all cases, a negative control ('kinetics buffer') is subtracted from each set of time-intensity curves. (a) Anti-H5 (anti-hemagglutinin) binding to H5 hemagglutinin. Analyte concentrations range from 0.47 – 30 nM (n=3). (b) CP-HIV-1/2 binding to HIV-1 p24. Analyte concentrations range from 0.39 – 25 nM (n=1). (c) NIH-3537 binding to HIV-1 p24. Analyte concentrations range from 0.39 – 100 nM (n=2). (d) Thermo Fisher Scientific Mouse anti-Human IgG Fc binding to human IgG. Analyte concentrations range from 0.47 – 30 nM (n=3). (e) Sigma Monoclonal Anti-Human IgG (Fc specific)-Biotin binding to human IgG. Analyte concentrations range from 14 – 900 nM (n=1). In each part, (i) shows the raw data (fractional binding over time) as dots, and fitted exponentials as lines; (ii) shows the Langmuir plots of fitted equilibrium values (fractional saturation values) against concertation. The fitted Langmuir curves are shown as solid lines; (iii) shows the observed rates k_{obs}, plotted against concentration. The solid lines show linear fits, where the gradients give k_{on} and the y-intercepts, k_{off}. In part (ci), two slightly different analyte concentration ranges are used (0.39 – 25 nM, and 1.6 – 100 nM) in order to cover a broader concentration range. The middle six concentrations (1.6 – 25 nM), are therefore repeated. These repeated points are used for the fit in (ciii).

Table S3 summarises the K_D, k_{on}, k_{off} values for video-µPADs.

Antigen	Antibody	Measured K_D (nM)	k_{on} (x10^5 $M^{-1}s^{-1}$)	k_{off} (x$10^{-3}s^{-1}$)
H5 hemagglutinin	Sino Biological Mouse Monoclonal Antibody to Influenza A Virus H5N1	2.9 (0.51)	1.8 (0.076)	0.81 (0.21)
HIV-1 p24	CP-HIV-1/2 [35]	5.1 (0.83)	1.2 (0.16)	2.4 (0.34)
HIV-1 p24	NIH-3537 [35,36]	8.2 (1.5)	0.49 (0.068)	0.91 (0.25)
Human immunoglobulin	Thermo Fisher Scientific Mouse anti-Human IgG Fc	1.2 (0.15)	1.1 (0.018)	0.16 (0.017)
Human immunoglobulin	Sigma Monoclonal Anti-Human IgG (Fc specific)-Biotin	170 (10)	0.10 (0.026)	2.8 (0.53)

Table S3. Summary of the video-µPAD fitted values for K_D, k_{on} and k_{off} for five different antibody-antigen pairs, following antibody concentration correction. Standard errors of the fitted values are shown in parentheses.

Association (k_{on}) and dissociation (k_{off}) rates

Comparison of k_{off}/k_{on} with K_D from Langmuir fit

The values of K_D extracted from the Langmuir fit are compared with the value of K_D obtained by dividing k_{off} by k_{on}, both extracted from the linear fit of k_{obs}. This tests how well the data fit the Langmuir model. Figure S8 shows that both the FortéBio and paper show a linear relationship between the two values for K_D. In both cases, k_{off}/k_{on} slightly overestimates the values compared to the Langmuir fits, although these differences are generally not significant, as shown by the 95% confidence interval boxes. Two points are significantly different from the y=x line at the 95% confidence level. This shows that the data fit the Langmuir model on paper and with the FortéBio Octet.

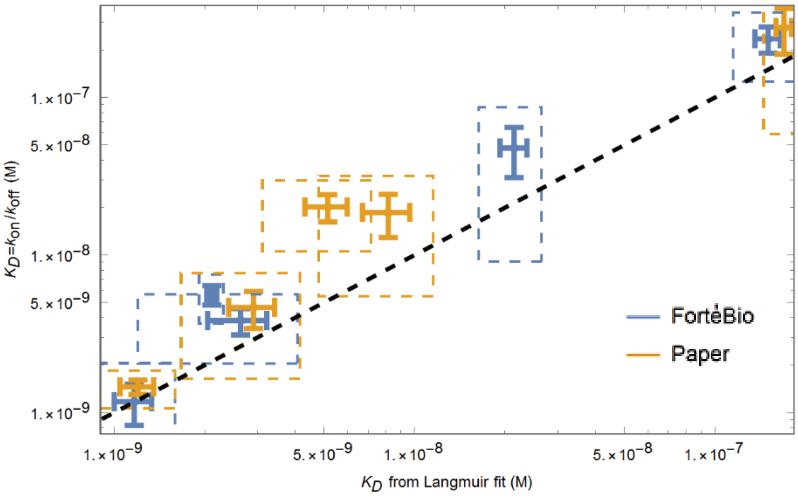

Figure S8. Comparison of K_D obtained from the Langmuir fit (on the x-axis) against K_D obtained by K_D=k_{on}/k_{off}, with k_{on} and k_{off} values obtained from k_{obs} linear fit. Values are shown for both paper and FortéBio Octet. The error bars give the standard error for the fitted parameters and the dotted boxes show the 95% confidence intervals for the fitted parameters. The black dotted line shows the function y=x for reference.

k_{on} and k_{off} measured on video-µPADs and FortéBio Octet

Figure S9 shows the comparison of k_{on} and k_{off} values measured on the FortéBio Octet and on paper. It can be seen that they follow the same trend, with values on paper generally higher than their corresponding values on the FortéBio Octet. k_{on} values generally increase, and k_{off} values slightly decrease with decreasing K_D on both platforms, as expected ($K_D = \frac{k_{off}}{k_{on}}$).

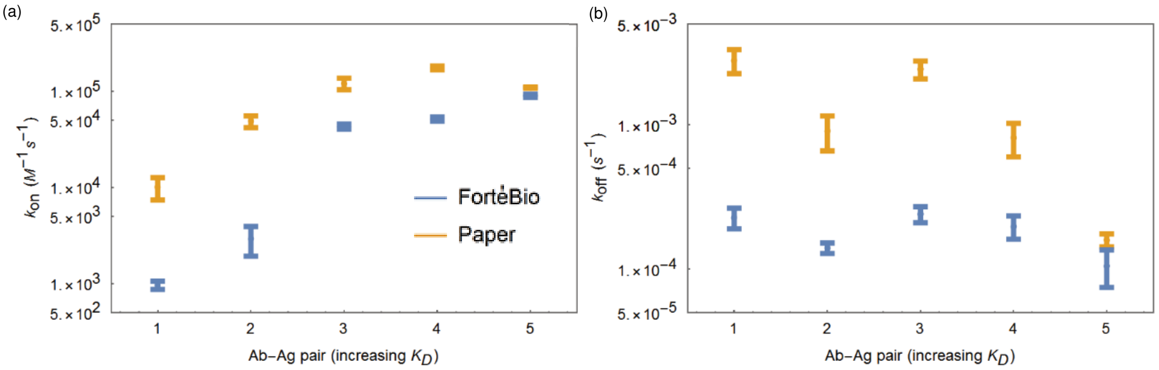

Figure S9. Comparisons of k_{on} (a) and k_{off} (b) measured on the FortéBio Octet and on paper for different antibody-antigen pairs, ordering in decreasing K_D (as measured on paper). The error bars represent the standard errors for the fitted parameters.

Comparison of video-µPAD measurements with camera and smartphone

The LG Nexus 5 smartphone is compared to the Canon Powershot G15 camera for the obtention of K_D values. An experiment measuring three sets of strips to quantify the binding of Thermo Fisher Scientific Mouse anti-Human IgG Fc to human IgG is filmed simultaneously using the two devices. The resulting raw data and exponential fits are shown in Figure S10a and b. Fitted infinite-time intensity values for each device are plotted against each other in Figure S10c. The Pearson Correlation Coefficient is 0.99, with a p-value of 1.9 x10^{-7}.

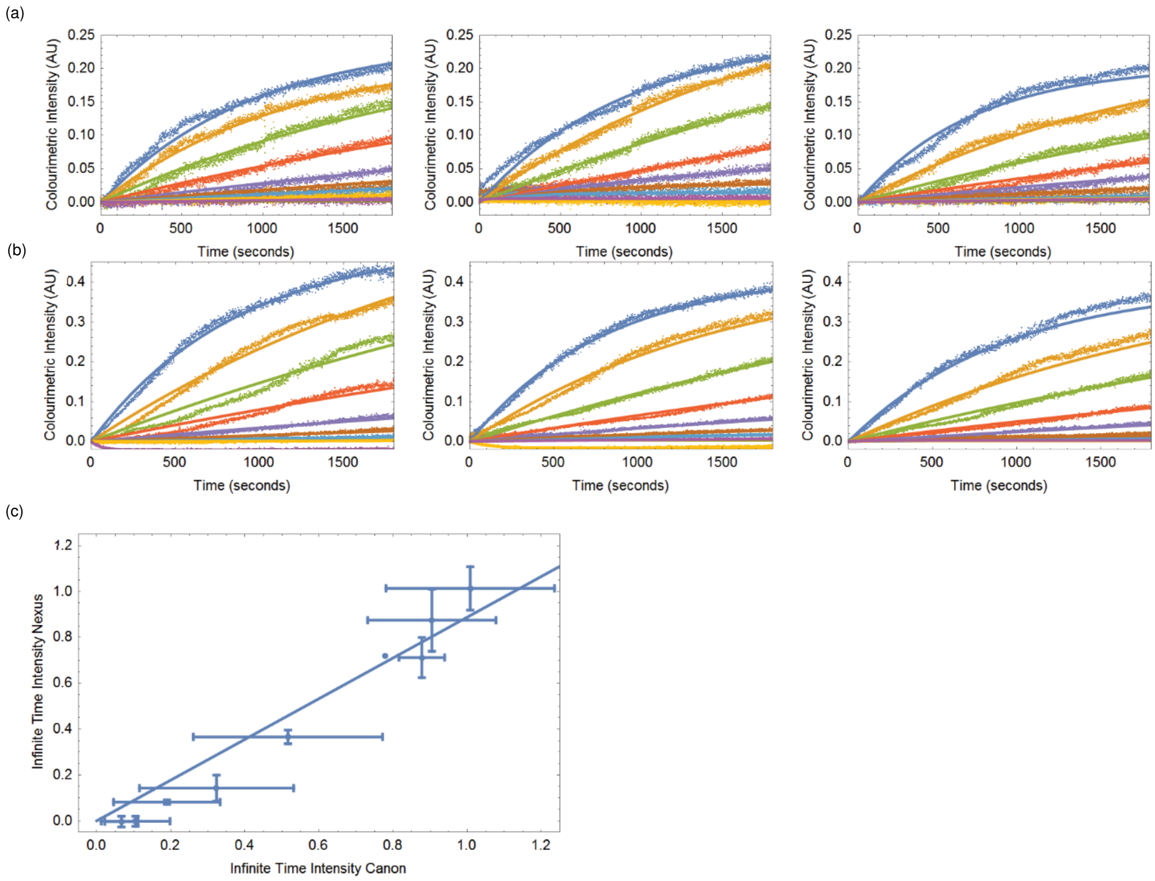

Figure S10. Video-µPAD measurements using Canon Powershot G15 and LG Nexus 5. (a) Triplicates of Canon camera measurements (dots), and exponential fits (solid lines). (b) Triplicates of Nexus 5 smartphone measurements (dots) and exponential fits (solid lines). (c) Fitted infinite-time intensity values plotted for one device against the other. The solid line shows a linear regression. Fitted line gradient is not significantly different from 1 (95% confidence intervals: 0.749-1.03; adjusted R^2 = 0.966). In parts (a) and (b), the time-scales for the datasets vary, so for clarity here, they are all cut to 1800 seconds. However, the fits are performed on the full datasets (up to 3600 seconds).

Multiplexing video-μPADs

To show that the antibody-antigen orientation is reversible for multiplexing (antigen on AuNPs and antibody on strip), all ten spots from the multiplexing proof-of-concept video-μPADs are simultaneously fitted to a Langmuir, shown in Figure S11. The resulting K_D value of 0.13 nM (4.1 nM corrected), is not significantly different to that for the same pair (CP-HIV-1/2 binding to HIV-1 p24), with a p-value of 0.25 from a two-tailed t-test (t-value = 1.1, degrees of freedom = 96).

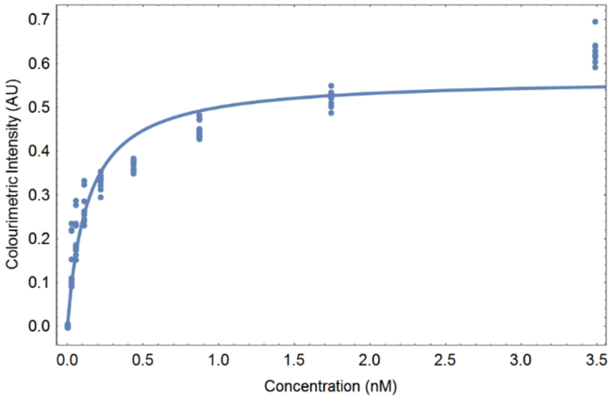

Figure S11. Reversed antibody-antigen orientation for multiplexing. This plot shows the infinite-time intensity values for all ten spots from the multiplexing proof-of-concept video-μPADs run at eight concentrations of antibody-functionalised AuNPs (0.027 – 3.5nM), and a blank of buffer only. All ten spots are simultaneously fitted to a Langmuir, giving K_D=0.13 nM.

Supporting Discussion: Assumptions K_D and Rate Limiting Factors

In this work, we fit our data to the Langmuir Adsorption Isotherm model, demonstrating strong agreement between our raw data and the 1:1 binding model. A number of assumptions are made to apply the model. Firstly, the nitrocellulose surface, a three-dimensional porous structure, is assumed to act as an 'ideal' flat surface, because the size of the pores (~μm) is significantly larger than the Ab-AuNP complexes (~10s of nm). Secondly, we assume that binding sites on the nitrocellulose strip are identical to each other and those on the AuNPs are identical to each other. Thirdly, the Langmuir model assumes a constant flow rate, high enough that there is no local solution analyte depletion, reducing the concentration near the surface. We demonstrate that the flow rate follows the Washburn equation[3] during "wet-out flow"

(Figure S3-S5), then Darcy's law[3] during "fully-wetted flow" (Figure S2), and observe a constant flow rate, once the strip is fully wetted, of 1.4 µL/minute.

In order to test the hypothesis that the reactions are limited by the antibody-antigen immunoreactions, rather than mass transport (advection or diffusion), characteristic times proposed by Berli et al.[4] are calculated.

The residence time, the time the analyte is in the capture zone, is given by $t_r = V_L/Q$, where t_r is the residency time, V_L is the volume of the capture zone, and Q is the volumetric flow rate.

The diffusive time, a characteristic diffusion time constant, is given by $t_D = d^2/D$, where t_D is the diffusive time, d is the pore diameter and D is the molecular diffusion coefficient,

The kinetic time, the characteristic time for immunoreactions, is given by $t_k = (k_{on}C)^{-1}$ where t_k is the kinetic time, and k_{on}, observed rate and C, concentration are typical values for an immunoreaction.

The calculated values are shown in Table S4 for Millipore HF180:

**Parameter**	**Value**
Q (µL/min)	1.43
Q (m^3/s)	2.38×10^{-11}
Capture zone height (m)	1.80×10^{-04}
Capture zone width (m)	3.00×10^{-03}
Capture zone length (m)	1.00×10^{-03}
V_L	5.40×10^{-10}
**t_r (s)**	**22.7**
**t_D (s)**	**0.1 – 1**
Typical k_{on} $(Ms)^{-1}$	6.00×10^{6}
Typical C (M)	1.30×10^{-10}
**t_k (s)**	**1.28×10^{3}**

Table S4. Characteristic time calculations for Millipore HF180. Using the equations proposed by Berli et al.[4], characteristic times for residency time, t_r, diffusive time, t_D, and kinetic time, t_k are calculated. Final characteristic times are shown in bold. t_k is ~57 time higher than t_r, suggesting that the reaction rate is limited by the binding of reagents, rather than advection or diffusion, so the Langmuir model can be applied.

These calculations show the kinetic time, t_k, is the slowest time by ~57 times for HF180 nitrocellulose (a factor of ~42 times is calculated for HF240). The diffusive time, the characteristic diffusion time in µPADs, is also much faster than the kinetic time, suggesting that the rate-limiting factor is protein complexation, rather than advection or diffusion, so the Langmuir model can be applied. Finally, we assume that evaporation is negligible over the relevant time-scales. We demonstrate (Figure S2) that this assumption is correct within our laboratory settings, leading to a constant reservoir concentration.

[1] E. Fu, S. A. Ramsey, P. Kauffman, B. Lutz, P. Yager, *Microfluid. Nanofluidics* **2011**, *10*, 29–35.

[2] E. W. Washburn, *Phys. Rev.* **1921**, DOI 10.1103/PhysRev.17.273.

[3] E. Fu, S. A. Ramsey, P. Kauffman, B. Lutz, P. Yager, *Microfluid. Nanofluidics* **2011**, *10*, 29–35.

[4] C. L. A. Berli, P. A. Kler, *Microfluid. Nanofluidics* **2016**, *20*, DOI 10.1007/s10404-016-1771-9.
