# Supplementary figures and images for: Quantifying Biomolecular Binding Constants using Video Paper Analytical Devices
Source: Chemistry. 2018 Jun 8;24(39):9783–7. doi: 10.1002/chem.201802394 (PMC6055620; doi:10.1002/chem.201802394)

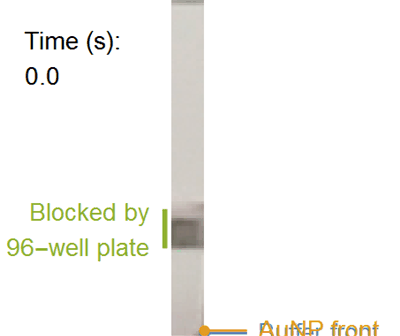

Supplement: Supplementary file 2 — Supplementary [file CHEM-24-9783-s002.gif]
